# Supplementary material for: Burden and clinical impact of ‘neglected’ transfusion-transmitted infections in Cameroon: A systematic review and meta-analysis
Source: PLoS Negl Trop Dis. 2026 May 26;20(5):e0014378. doi: 10.1371/journal.pntd.0014378 (PMC13221143; doi:10.1371/journal.pntd.0014378)
Supplement: S1 Fig — (DOCX) [file pntd.0014378.s003.docx]

**S1 Fig**. **Subgroup analysis, sensitivity analysis, and publication bias of studies on malaria in blood donors.**

*In this supplemental file, subgroup analysis is presented to evaluate the impact of confounding variables (e.g., study area) on the pooled estimates of transfusion-transmitted malaria proportion. Graphical representations (forest plots) are presented below. We have also performed a publication bias analysis (funnel plot) coupled with statistical analysis (Egger test).*

- *Subgroup analysis of the pooled transfusion-transmitted Plasmodium proportion by year of data collection*

*
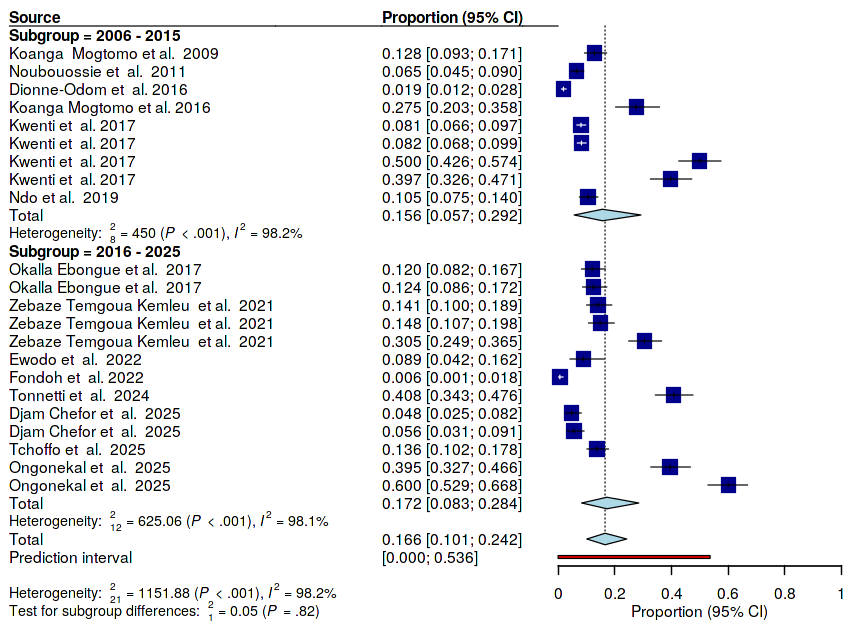
*

- *Subgroup analysis of the pooled transfusion-transmitted Plasmodium proportion by areas*


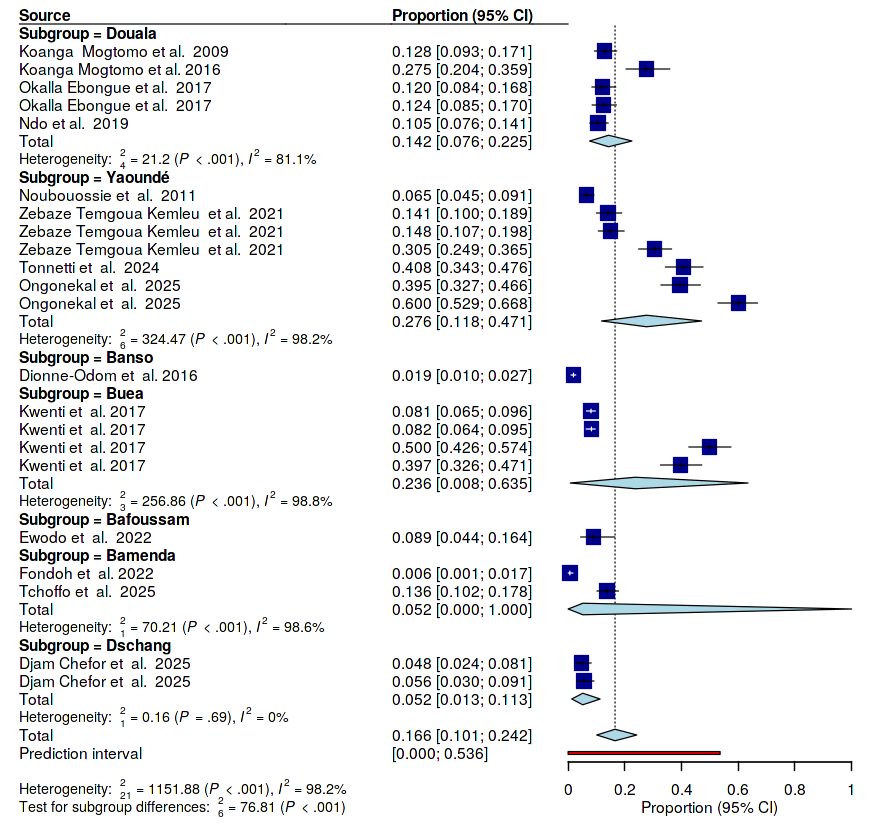


- *Subgroup analysis of the pooled transfusion-transmitted Plasmodium proportion by regions*


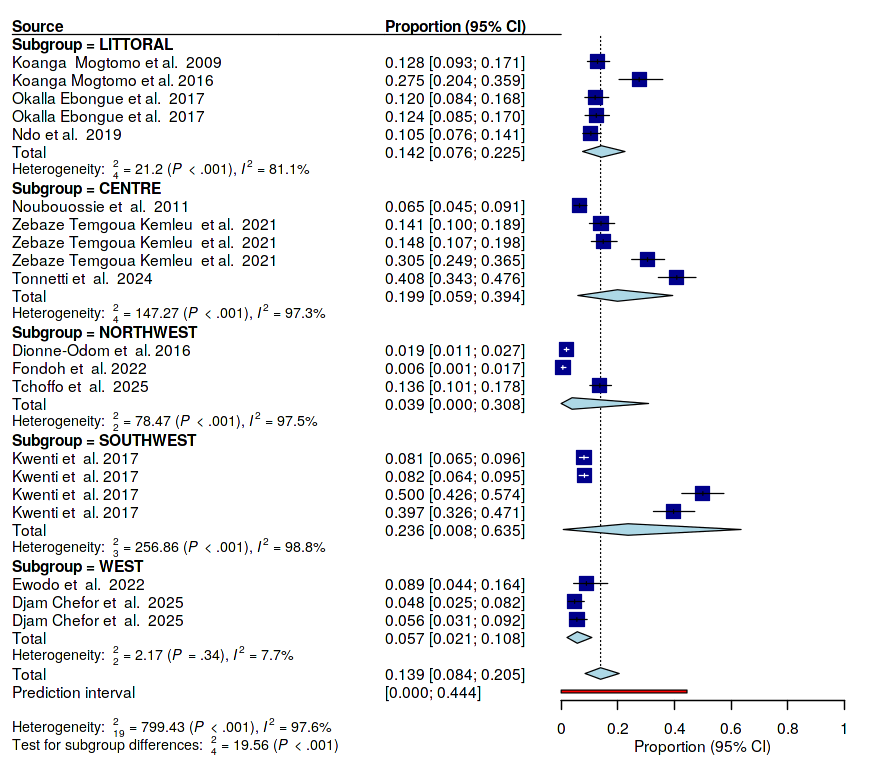


- *Subgroup analysis of the pooled transfusion-transmitted Plasmodium proportion by settings*


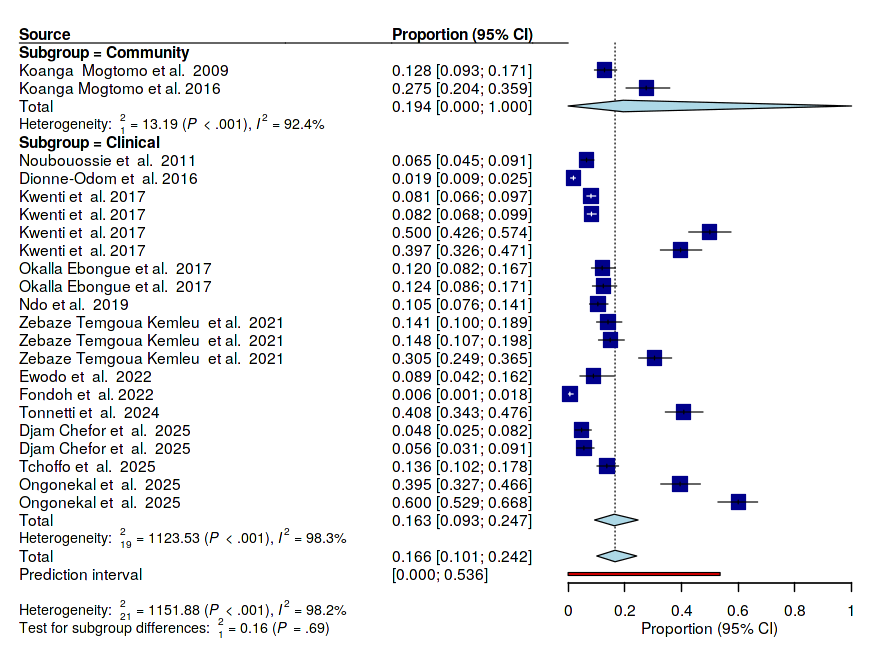


- *Subgroup analysis of the pooled transfusion-transmitted Plasmodium proportion by testing assays*


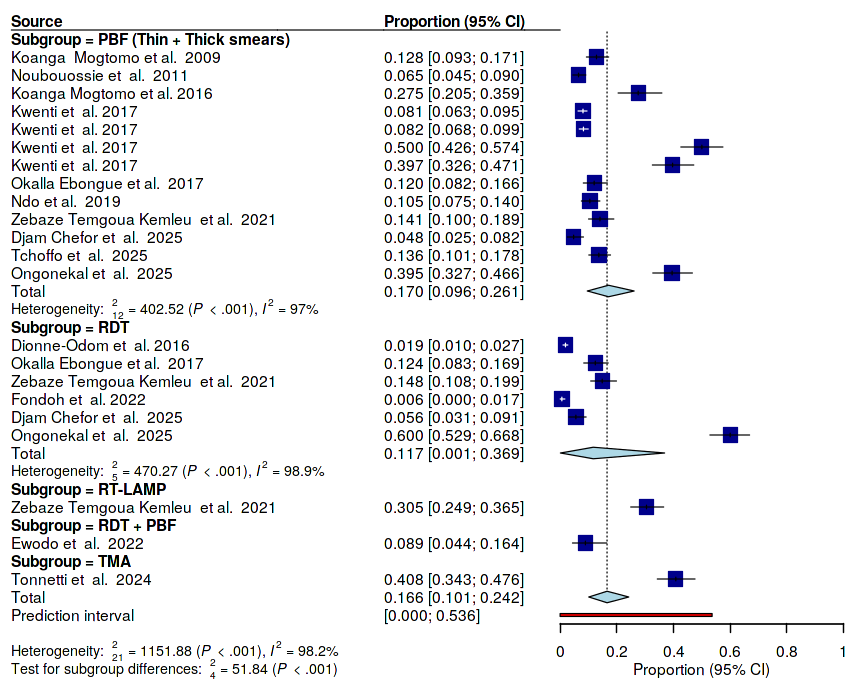


- *Subgroup analysis of the pooled transfusion-transmitted Plasmodium proportion by sample size*


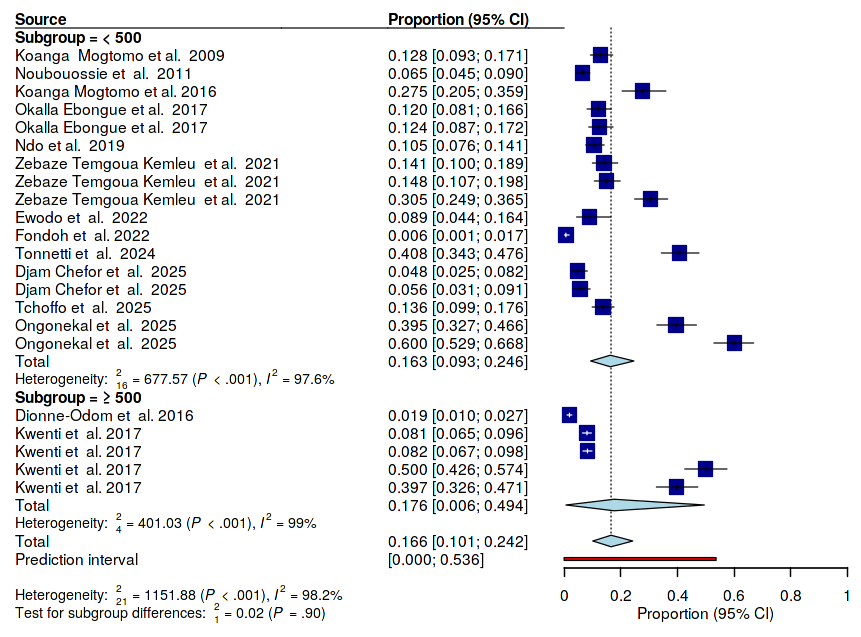


- *Publication bias*


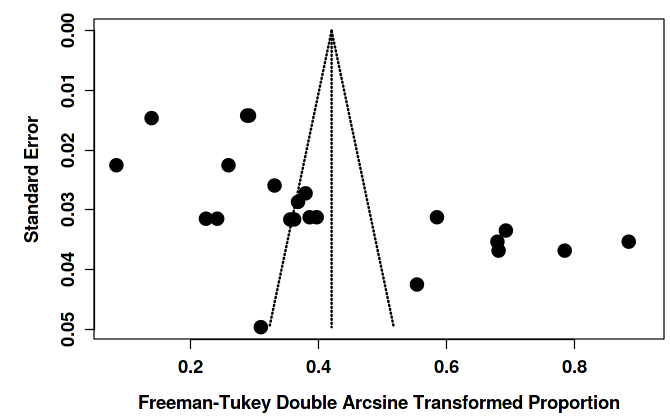


***Note*.** The funnel plot indicates a potential publication bias. The Egger's test supports the presence of funnel plot asymmetry (intercept: 10.85, 95% CI: 4.4 - 17.31, t: 3.294, *p* = 0.004).

**References (studies included for systematic review and meta-analysis)**

1. Koanga Mogtomo ML, Louandji Fomekong S, Fotso Kuate H, Ngono Ngane RA. Détection des agents infectieux dans les banques de sang de Douala (1995-2004). Cah Santé. 2009;19: 1–8.

2. Noubouossie D, Tagny CT, Same-Ekobo A, Mbanya D. Asymptomatic carriage of malaria parasites in blood donors in Yaoundé. Transfus Med. 2012;22: 63–67. doi:10.1111/j.1365-3148.2011.01121.x

3. Dionne-Odom J, Mbah R, Rembert NJ, Tancho S, Halle-Ekane GE, Enah C, et al. Hepatitis B, HIV, and syphilis seroprevalence in pregnant women and blood donors in Cameroon. Infect Dis Obstet Gynecol. 2016;2016: 4359401. doi:10.1155/2016/4359401

4. Koanga Mogtomo ML, Kojom Foko LP, Assokom Okoubalimba EV, Embolo Enyegue E, Ngono Ngane AR. High risk of transfusion-transmitted malaria (TTM) from student blood donors living in the town of Douala, Cameroon. J Clin Infect Dis Pract. 2016;1: 1. doi:10.4172/2476-213x.1000108

5. Kwenti TE, Njunda LA, Tsamul B, Nsagha SD, Assob Nguedia JC, Tufon KA, et al. Comparative evaluation of a rapid diagnostic test, an antibody ELISA, and a pLDH ELISA in detecting asymptomatic malaria parasitaemia in blood donors in Buea, Cameroon. Infect Dis Pov. 2017;6: 103. doi:10.1186/s40249-017-0314-2

6. Okalla Ebongue C, Ngouadjeu Dongho E, Texier G, Nda Mefo’o J-P, Sume Etapelong G, Ayong L, et al. Residual risk of transfusion-transmitted malaria infection in a malaria endemic sub-Saharan African setting. Transl Med Commun. 2017;2: 4. doi:10.1186/s41231-017-0013-9

7. Kengne M, Tsata DCW, Ndomgue T, Nwobegahay JM. Prevalence and risk factors of HTLV-1/2 and other blood borne infectious diseases among blood donors in yaounde central hospital, Cameroon. Pan Afr Med J. 2018;30: 125. doi:10.11604/pamj.2018.30.125.14802

8. Ndo C, Ewane Ngalle TJ, Ewodo S, Olemba C, Adiogo D. Hepatitis B and C, HIV, syphilis seroprevalences and asymptomatic carriage of hemoparasites among blood donors at the Douala General hospital in Cameroon, Central Africa. J Sci Tech Res. 2019;18: 13968–13974. doi:10.26717/BJSTR.2019.18.003227

9. Tchuandom SB, Lissom A, Ateba GHM, Tchouangueu TF, Tchakounte C, Ayuk AR, et al. Dengue virus serological markers among potential blood donors: An evidence of asymptomatic dengue virus transmission in Cameroon. Pan Afr Med J. 2020;36: 14. doi:10.11604/pamj.2020.36.185.22128

10. Kemleu SGZT, Ngando L, Nguekeng E, Fogang B, Kapen MM, Fopa SI, et al. Diagnostic performance of a rapid whole blood-based RT-LAMP method for malaria diagnosis among apparently healthy blood donors and febrile neonates in Cameroon. PLoS One. 2021;16: e0246205. doi:10.1371/journal.pone.0246205

11. Ewodo S, Yondo J, Divine A, Sonhafouo K, Olemba C, Sylvie N, et al. Impact of plasmodial parasitaemia on the quality of erythrocyte concentrates distributed at the blood transfusion Center of the Regional Hospital of Bafoussam-Cameroon. Am Acad Sci Res J Eng Technol Sci. : 58–67.

12. Fondoh VN, Ndzenjempuh N, Stella T, Fondoh RM, Awasom CN, Enow-Tanjong R, et al. Prevalence of alpha and beta haemolysin among blood group O donors in Bamenda, Cameroon. Afr J Lab Med. 2022;11: 1–6. doi:10.4102/AJLM.V11I1.1432

13. Medi CI, Voundi EV, Lobe SA, Eyoum Bille B, Ngogang MP, Ndoumba Mintya A, et al. COVID-19 in blood donors at Laquintinie hospital in Douala during the third wave: A cross sectional study. Open J Epidemiol. 2022;12: 367–379. doi:10.4236/ojepi.2022.123030

14. Ndoumba Mintya A, Yap BII, Bouopda R, Dimegni E, Kouongni Y, C TT, et al. Seroprevalence of anti-SARS-CoV-2 antibodies in blood donors at the Yaoundé University Teaching Hospital, Cameroon. J Sci Dis. 2024;1: 30–34.

15. Tonnetti L, Groves JA, Self D, Yadav MC, Tayou Tagny C, Rakoto Alson OA, et al. Estimated *Plasmodium* 18S ribosomal RNA prevalence in asymptomatic blood donors from three African countries. Vox Sang. 2024; 13756. doi:10.1111/vox.13756

16. Djam Chefor A, Djienang ELM, Tabah EN, Ndongmo Donjio CL, Motangu B, Ifang S, et al. Prevalence of malaria among blood donors and recipients in Dschang health district, West Cameroon. Int J Res Reports Hematol. 2025;8: 14–23. doi:10.9734/ijr2h/2025/v8i1161

17. Tchoffo FH, Tofel HK, Abongwa LE, Ndifor RF, Ntonifor NH. Assessing the impact of haemoparasites on albumin and IgE blood levels in transfused blood at the Regional Hospital Bamenda, North West Cameroon. Int J Trop Dis Health. 2025;46: 28–39.

18. Ongonekal G, Gueguim C, Sipewa M, Abange B, Biock C, Ndeme C, et al. Comparative study of two malaria diagnostic techniques in the context of transfusion safety in blood donors at the Yaoundé University Hospital. Cent African J Public Heal. 2025;11: 186–191. doi:10.11648/j.cajph.20251104.14
