# Supplementary material for: Burden and clinical impact of ‘neglected’ transfusion-transmitted infections in Cameroon: A systematic review and meta-analysis
Source: PLoS Negl Trop Dis. 2026 May 26;20(5):e0014378. doi: 10.1371/journal.pntd.0014378 (PMC13221143; doi:10.1371/journal.pntd.0014378)
Supplement: Attachment — Submitted filename: Response to Reviewers.docx [file pntd.0014378.s004.docx]

**Response to Reviewers**

**Date: 20.04.2026**

**Manuscript title**: Burden and clinical impact of ‘neglected’ transfusion-transmitted infections in Cameroon: a systematic review and meta-analysis

**Manuscript ID**: PNTD-D-26-00009

**Journal**: PLOS Neglected Tropical Diseases

**Article type**: Systematic Review and Meta-analysis

Dear Journal Editorial Office,

In this letter, we try to respond to each point raised by the Editors and reviewers.

We thank the editor and reviewers for their comments about the paper. We are very thrilled with the revised version of the paper.

Please find below the answers (in brown) to each point raised by the reviewers. Revisions are highlighted in yellow in the revised manuscript.

**Additional Editor Comments:**

Dear Dr. Foko,
Thank you for submitting your manuscript for review to PLoS NTD. Although you have addressed an important public health issue in Cameroon, the manuscript has significant issues related to description of data, limitations, and analysis of the underlying studies. In addition to the issues raised by the reviewers, please address the following:

1. On line 212-213, you state that “94.4% of the studies failed to provide site details (i.e., related to blood transfusion), and 66.7% did not use an appropriate statistical analysis” with the study details provided in S2 Table.

a. This is a significant issue as it raises questions about the conclusion drawn and the information presented in Table 1. Please move the column on site details and appropriate statistical analysis from S2 Table to Table 1.

**Reply**: We understand this point, and have moved these two columns (Table 1 R1).

b. Please provide appropriate descriptive titles for the tables.
**Reply**: Done (Table 1 R1).

c. The limitations section of your manuscript lacks detail and does not attribute limitations to various factors addressed in S2 Table. The limitations section needs to be re-written.

**Reply**: We have added this point (Lines 441-464).

2. For proportions provided, e.g., line 247 for pooled estimates of Plasmodium infection 15.6%, please provide the numerator and denominator in addition to the percentage and 95% CI.

**Reply**: We have added this point (Lines 292-298).

3. Table 1 shows the donor selection criteria for all donors together (family, voluntary, and paid). The three categories have different risk profiles as blood donors with respect to TTIs. Although there is limited data, does the risk profile vary for these three groups regarding Malaria transmission e.g., paid donors may not have access to Malaria preventive measures, and therefore would be a higher risk? Is it possible to evaluate the three groups separately based on the available literature?

**Reply**: We have added this interesting point (Lines 375-384). Thank you

4. Although Plasmodium parasite was the major pathogen based on your inclusion criteria for publications, please provide data on HIV, HCV, and HBV, as it will help readers understand the scope of the issue better when compared to viral TTIs. Viral TTI data may not necessarily be an analysis of excluded publication but from existing literature.

**Reply**: We have added this point (Lines 357-361).

5. Malaria transmission varies by season. Transmission may have also declined due to control programs.

a. Based on the limited number of publications, please address how these two factors impact your study.

b. Additionally, considering that the donors and recipients reside in a Malaria endemic area, you have not addressed the possibility that recipients may have tested positive due to transmission by a mosquito bite rather than by blood transfusion from a false negative tested donor. From the selected literature, please describe how traceback studies were performed to attribute blood donors to recipients testing positive for Plasmodium.
Significant confounding factors exist and may have been addressed in the selected studies, however, these should also be addressed, either in the discussion section or in the limitations section of your manuscript.

**Reply**: We have added this point (Lines 441 – 450 & Lines 455-464).

6. Please consider providing a map of Camerron along with Table 1 so it is easier for readers to visualize where the studies were performed. What proportion of the country (area and population) is covered by the analysis performed? This is an important factor to analyze considering the impact on public health policy of your manuscript.

**Reply**: We have added a map of Cameroon showing the regions where studies were conducted. We have also discussed the generalizability of the findings in the limitations (Lines 441 – 443).

**Reviewer #1**:

**Introduction**

Line 47: Please indicate all four viral and bacterial pathogens
**Reply**: Done (Lines 47-48).

Line 83: Please indicate the duration of blood donations recorded by WHO
**Reply**: Done (Lines 101-103).

Line 101: Please indicate regions where priority is given for 4 pathogens described.
**Reply**: Done (Lines 119- 120).

Line 108-109: Please indicate the location of health facilities and provide specifics about countries implementing national blood safety policies and surveillance
**Reply**: Done (Line 126).

Line 115: Please describe in 1-2 sentences on what aspects the mentioned pathogens are being neglected in developing countries.
**Reply**: Done (Lines 128-131).

**Material Methods**

Line 128: Though authors stated no ethical authorization is required for the current study, please indicate if any ethical considerations were followed in your protocol while handling the data from previous literature.
**Reply**: Done (Lines 148-151).

Line 161: Please indicate whether the three reviewers (mentioned in line 156) and three authors (mentioned in Line 161) are same.
**Reply**: Done (Line 192).

Line 167: Please indicate how many reviewers assessed methodological quality of each study and whether they worked independently.
**Reply**: Done (Lines 199-200).

Line 169: Clarify the sentence structure. Findings from the included studies or SRMA of the included studies? Expand the abbreviation SRMA and indicate that the SRMA mentioned is the current study.
**Reply**: Done (Line 202). The term ‘SRMA’ has already been expanded in the introduction (Lines 138-139).

**Results**

Line 195: Please indicate the dates of data collection for identified records
**Reply**: Done (Line 229).

Line 199: Please clarify whether you identified two or three papers after inspection of bibliographies.
**Reply**: Corrected (Line 233). Three additional studies were identified.

Line 211: Please indicate all four criteria for which bias risk of the studies was good.
**Reply**: Done (Lines 246-248).

**Discussion**

Line 297: Please indicate the pooled estimates of major TTIs in Cameroon for comparison. Please clarify whether the statement is true for T. gondii infections. You did not provide a pooled estimate for T. gondii infection in Results section.
**Reply**: Done (Lines 273-274 & Lines 357-361).

Line 300: Please indicate the non-malarial pathogens that you are discussing.
**Reply**: Done (Line 362).

Line 301: So far, there is no empirical evidence presented for SARS-CoV-2 causing transfusion-transmitted infections. Therefore, discussing SARS-CoV-2 with relevant to TTI is not necessary. Please remove this sentence.
**Reply**: Removed (Line 373).

Line 317: Please elaborate why deletions in the histidine-rich protein 2 genes are important for false negative results in RDTs.
**Reply**: Done (Lines 390-396).

Line 321: Please elaborate how significant work-related pressure faced by personnel at blood banks in Cameroon affect the likelihood of additional testing for malaria in blood donors.
**Reply**: Done (Lines 399-404).

**Conclusion**

Line 356: please clarify how T. gondii prevalence outnumber that of major TTIs.
Supporting information
S1 Table, Introduction, Objectives, item # 4: Please revise as “Introduction, 3rd and 4th paragraph” under the location where item is reported
**Reply**: We are not able to address this comment, as only one study, included in the present review, tracked *T. gondii* in blood donors.

Figures
Figure 1: Please remove SARS-CoV2 from the data as there is no empirical evidence presented for this pathogen causing transfusion-transmitted infections.
**Reply**: We prefer to keep this virus in the analysis given the yet dark areas on the understanding of the natural history of this virus. We have adjusted the text and figures accordingly (Lines 63-64, Lines 178-183, Lines 281-283).

Table S1: Item#26, under location where item is reported, please revise the title to “Competing interests”. Item#27, under location where item is reported, please revise the title to “Data availability statement” and indicate that it is not applicable.

**Reply**: Corrected.

**Reviewer #3: General comments**

1) While this meta-analysis is constrained by a limited number of eligible studies, the scarcity of data is itself a significant finding. Further, as mentioned by the authors, as the available research is restricted to three regions of Cameroon, the results may not be fully generalizable to the national level. These limitations are addressed in the discussion; however, documenting this regional data gap is a primary contribution of this report and warrants publication.

**Reply**: Dear colleague, we have added this important point raised by you (Lines 348-352). Thank you

2) Considering the limitations of the studies, the authors identified challenges which should be addressed to improve blood safety in Cameroon. These include awareness of neglected diseases among healthcare providers and policymakers, diagnostic and logistical constraints, and low grade infections.

**Reply**: You are right. All these points have been addressed in the paper.

The authors should address the following comments in their revised manuscript:

3) Line 65: Define the abbreviation “TTIs” in the abstract

**Reply**: Done (Lines 66-67).

4) Line 152: Please explain why publications prior to 2006 were excluded from this analysis.

**Reply**: Done (Lines 175-178).

5) Line 208: Please clarify the meaning of “accidentally found” in the context of the 2 published studies.
**Reply**: We have clarified this point (Line 242). The detection of this parasite was not included in the predefined study protocols.

6) Lines 254-263: Please discuss the impact of these co-infections on blood safety.

**Reply**: We have added this point (Lines 413-423). Thank you

7) Lines 313-324: While this paragraph underscores the operational challenges and limited efficacy associated with RDT and PBF testing, a clear statement regarding the authors' proposed solution is currently missing. Explicitly detailing these improvements would clarify the study's contribution.

**Reply**: We have added this point (Lines 406-411).

8) Line 322: Please briefly explain the “significant work-related pressure” experienced by personnel at blood banks in Cameroon.

**Reply**: Done (Lines 399-404).

Thank you for these comments

Regards

**Dr Loick P. Kojom Foko, PhD**

CEREBA, Douala-Cameroon

The University of Douala, Cameroon

E-mail: [kojomloick@gmail.com](mailto:kojomloick@gmail.com)
